# P-gp and taxanes

**DOI:** 10.18632/oncoscience.56

**Published:** 2014-06-19

**Authors:** Jeroen J.M.A. Hendrikx, Jos H. Beijnen, Alfred H. Schinkel

**Affiliations:** Department of Pharmacy & Pharmacology, The Netherlands Cancer Institute/ Slotervaart Hospital, Amsterdam, The Netherlands

In the past years, the development of oral formulations of the taxane anticancer drugs paclitaxel (Taxol^®^) and docetaxel (Taxotere^®^) has been the focus of preclinical and clinical research in our groups. A major limitation in the concept of oral administration of paclitaxel and docetaxel is their low oral availability [1]. Paclitaxel and docetaxel have poor aqueous solubility and upon oral administration, intestinal uptake is seriously hampered by drug efflux through the active efflux transporter P-glycoprotein (P-gp/MDR1/ABCB1) and by drug metabolism via Cytochrome P450 (CYP) 3A [2,3]. Several studies by our groups have shown that the oral bioavailability of paclitaxel and docetaxel can be enhanced by 1) oral administration of a solid dispersion of these taxanes [4], and 2) combining taxanes with P-gp inhibitors or CYP3A4 inhibitors to reduce intestinal and hepatic drug efflux transport and drug metabolism [1]. Importantly, since P-gp is not only expressed in tissues like intestine, liver, and kidney, but also at the blood-brain barrier (BBB) where it keeps its substrates out of the brain [5], co-administration of orally administered taxanes with P-gp inhibitors might also result in increased brain penetration of the taxanes.

Enhanced brain penetration of anticancer drugs due to P-gp inhibition at the BBB can be a double-edged sword: on the one hand, for specifically targeted anticancer drugs, including many tyrosine kinase inhibitors (TKIs), with a low chance of causing problems in the central nervous system (CNS), it may comparatively safely enhance efficacy of the drugs against brain tumor parts or brain (micro)metastases that are positioned behind a functionally intact BBB. On the other hand, for potentially neurotoxic cytotoxins such as the taxanes, there is a risk of disrupting brain functions upon increased CNS penetration, which may thus be undesirable. Our recent study was set up in part to assess the risk of enhancing taxane brain penetration when applying oral taxane administration regimens.

We first examined whether we could substantially increase the oral availability of taxanes (at 10 mg/kg) in mice by simultaneous inhibition of P-gp and CYP3A4 using oral co-administration of elacridar (25 mg/kg) and ritonavir (12.5 mg/kg), and to what extent this would affect P-gp transport at the blood-brain barrier [6]. Comparison of the taxane plasma levels obtained in our study with previously reported data obtained from oral administration of taxanes to knockout mice [2,3] showed that orally administered elacridar and ritonavir at comparatively low doses could completely (for paclitaxel), or almost completely (for docetaxel) inhibit intestinal and hepatic P-gp and CYP3A4 activity. We also demonstrated that co-administration of the taxanes with elacridar and ritonavir simultaneously (instead of only with elacridar or only with ritonavir) resulted in a further increase in plasma levels of the taxanes [6]. In contrast, relative brain penetration of the taxanes after oral administration was not affected after “boosting” the taxane plasma levels with oral elacridar. Even at the highly increased plasma concentrations of taxanes (~1000 ng/mL) obtained after boosting with both elacridar and ritonavir, relative brain accumulation was still very similar to that seen after boosting with elacridar, or even after oral administration of taxanes without a booster [6].

These results seemed to contrast with previously reported data on increased relative brain penetration after intravenous administration of paclitaxel (10 mg/kg) with orally administered elacridar (25 mg/kg) to mice [7]. Interestingly, this increased relative brain penetration was only observed during the first hours after intravenous administration, when plasma concentrations were high, exceeding 1000 ng/mL, but not at later time points when lower plasma paclitaxel concentrations prevailed. It is possible that, at the initially high plasma concentrations after intravenous administration, P-gp-mediated transport of paclitaxel is operating close to its saturation level. Under those circumstances, P-gp at the BBB will be more sensitive to partial inhibition than at lower plasma paclitaxel concentrations [8]. Another explanation might be that one or more other efflux transporters of as yet unknown identity are involved in paclitaxel efflux across the BBB with lower K_m_s than P-gp. At low plasma concentrations of paclitaxel, these transporter(s) will contribute substantially to limiting brain accumulation of paclitaxel. Partial inhibition of P-gp by elacridar will then have much less of a noticeable impact. At higher plasma concentrations of paclitaxel, the transport capacity of the other transporter(s) will be saturated and partial inhibition of P-gp will thus have more of an impact. Although at this moment in time we can only speculate on the main underlying mechanism, it is likely that the initially far higher plasma concentrations after intravenous compared to oral administration of paclitaxel caused the difference in brain penetration of orally and intravenously administered paclitaxel when co-administered with the P-gp inhibitor elacridar.

In conclusion, we believe that co-administration of orally applied taxanes and P-gp inhibitors at low dose may provide a relatively safe strategy to boost plasma exposure of taxanes in patients, as relative brain exposure is unlikely to be higher than that in the currently used intravenous schedules. Furthermore, our data suggest that one should be cautious in predicting drug-drug interactions at the BBB after oral administration of a substrate drug based on data obtained after intravenous administration; especially potentially prominent effects of differences in plasma concentrations of the drugs between the different administration routes should be taken into account.
